# Use of Muscle Ultrasonography in Morphofunctional Assessment of Amyotrophic Lateral Sclerosis (ALS)

**DOI:** 10.3390/nu16071021

**Published:** 2024-03-31

**Authors:** Juan J. López-Gómez, Olatz Izaola-Jauregui, Laura Almansa-Ruiz, Rebeca Jiménez-Sahagún, David Primo-Martín, María I. Pedraza-Hueso, Beatriz Ramos-Bachiller, Jaime González-Gutiérrez, Daniel De Luis-Román

**Affiliations:** 1 Servicio de Endocrinología y Nutrición, Hospital Clínico Universitario de Valladolid, 47003 Valladolid, Spainbeatriz.ramos.bachiller@gmail.com (B.R.-B.);; 2 Centro de Investigación en Endocrinología y Nutrición, Universidad de Valladolid, 47003 Valladolid, Spain; 3 Facultad de Medicina, Universidad de Valladolid, 47003 Valladolid, Spain; 4 Servicio de Neurología, Hospital Clínico Universitario de Valladolid, 47003 Valladolid, Spain

**Keywords:** muscle ultrasonography, amyotrophic lateral sclerosis, morphofunctional assessment, bioimpedanciometry

## Abstract

Amyotrophic lateral sclerosis (ALS) is a progressive disease with a high prevalence of malnutrition that can influence prognosis. The main objective of this study is to compare the validity of muscle ultrasonography in the diagnosis of malnutrition and the prognosis of patients with ALS. Methods: This is a prospective observational study that analyzes the nutritional status of patients at the beginning of nutritional monitoring. The morphofunctional assessment included the examination of anthropometric variables such as weight, height, body mass index (BMI), arm circumference, and calf circumference. Additionally, electrical bioimpedanciometry (BIA) was used to measure electrical parameters and estimate other relevant metrics. Muscle ultrasonography^®^ (quadriceps rectus femoris (QRF)) assessed muscle mass parameters, including muscle area index (MARAI), anteroposterior diameter of the QRF (*Y*-axis) (cm), transverse diameter of the QRF (*X*-axis) (cm), and the sum of the quadriceps thickness (RF+VI) (cm), as well as muscle quality parameters such as echogenicity and the Y–X index. Results: A total of 37 patients diagnosed with amyotrophic lateral sclerosis (ALS) were included in this study. Of these patients, 51.4% were men. The mean age was 64.27 (12.59) years. A total of 54.1% of the patients had a bulbar onset of amyotrophic lateral sclerosis, and 45.9% had spinal onset. The percentage of subjects with malnutrition diagnosed by the Global Leadership Initiative on Malnutrition (GLIM) criteria was 45.9% of patients. There was a direct correlation between muscle mass parameters assessed by muscle ultrasonography (RF+VI) and active mass markers measured by bioimpedanciometry (body cellular mass index (BCMI) (r = 0.62; *p* < 0.01), fat-free mass index (FFMI) (r = 0.75; *p* < 0.01), and appendicular skeletal mass index (ASMI) (r = 0.69; *p* < 0.01)). There was a direct correlation between echogenicity and resistance (r = 0.44; *p* = 0.02), as well as between the fat-free mass index and the Y–X index (r = 0.36; *p* = 0.14). Additionally, there was a negative correlation between echogenicity and BCMI (r = −0.46; *p* < 0.01) and ASMI (r = 0.34; *p* = 0.06). Patients with low quadriceps thickness (male < 2.49 cm; female < 1.84 cm) showed an increased risk of hospital admission adjusted by age, sex, and presence of dysphagia (OR: 7.84 (CI 95%: 1.09–56.07); *p*-value = 0.04), and patients with low-quality mass (Y–X index < 0.35) had a higher risk of hospital admission adjusted by age, sex, and presence of dysphagia (OR: 19.83 (CI 95%: 1.77–222.46); *p*-value = 0.02). Conclusions: In patients with ALS, ultrasonography echogenicity was inversely related to BCMI, FFMI, and ASMI, and the Y–X index was directly related to FFMI. The lowest quartiles of quadriceps thickness and Y–X index are risk factors for hospital admission.

## 1. Introduction

Motor neuron diseases are a group of chronic, progressive neurodegenerative disorders that affect the central nervous system. The most frequent disease is amyotrophic lateral sclerosis (ALS), with an incidence of 2.8 new cases per 100,000 people in Europe and a prevalence of 5.4 cases per 100,000 people [1]. This disease can present symptoms such as muscle weakness, especially in the arms and legs, alterations in speech, swallowing, or breathing, as well as emotional problems [2]. These circumstances can lead to an increased risk of malnutrition at diagnosis in these patients, which can be observed in up to 46% of patients [3]. Malnutrition in patients with ALS can lead to an increased risk of mortality and complications. It has been observed that malnutrition determined by subjective global assessment (SGA) was an independent risk factor for mortality (HR: 4.6 (CI 95% 1.5–13.9)) [3]. Therefore, medical nutritional therapy (MNT) plays an important role in the management of these diseases since malnutrition, weight loss, and impaired muscle function can affect the quality of life of patients with ALS [4].

Nutritional evaluation is an important part of the diagnosis and monitoring of MNT. In patients with ALS, nutritional assessment is complex and usually limited to anthropometric parameters. It has been proposed to monitor nutritional treatment in relation to changes in body weight and body mass index [5]. However, these measurements can often be interfered with by multiple issues, such as functional impairment, and they do not adequately reflect changes in the patient’s body composition. 

In body composition assessment, some methods are limited to research due to their difficulty in accessing and performing (air-displacement plethysmography, in vivo neutron activation analysis, isotope dilution, and total body potassium count). There are also other techniques, such as computed tomography (CT) scanning and DEXA, but they are not commonly used due to technical difficulties. Finally, there are methods, such as bioelectrical impedanciometry and classical anthropometry, used in routine clinical practice due to their easy implementation, accessibility in consultation, and reproducibility [6]. The use of body composition assessment techniques associated with tests that evaluate functionality in nutritional assessment gives rise to a new concept: the morphofunctional assessment of nutritional assessment [7]. 

In this context, the use of muscle ultrasonography has proven to be a new dynamic alternative for the quantitative and qualitative assessment of muscle [8]. Ultrasonography has become a useful tool for nutritional assessment due to its ability to determine the depth of subcutaneous fat and lean body mass in a non-invasive manner. Simple equipment is used to perform this test, and with skilled hands, it can take little time. Studies have shown that ultrasonography has adequate reliability and validity for the assessment of nutritional status [9]. Therefore, muscle ultrasonography may be a promising technique in these patients since it quantifies changes in muscle structures during malnutrition and can provide information on functional changes. On the other hand, it is simple and can be performed in the consultation or bedside, avoiding unnecessary patient travel. 

In patients with ALS, the assessment of body composition is complex due to the technical difficulty in carrying it out due to the mobility and availability of the patient. Furthermore, the existing evidence is scarce due to the low prevalence of the disease. Nutritional ultrasonography can be a complementary tool for the diagnosis and monitoring of malnutrition. However, there are some limitations, such as clear measurement criteria and the choice of the most appropriate muscle group. A validation of this test is required in different pathologies with the establishment of normality values and the comparison with other methods with more experience and evidence [7]. 

The main objective of this study is to compare the usefulness of muscle ultrasonography in the diagnosis of malnutrition with respect to common techniques such as handgrip strength and impedanciometry in patients with ALS. Our hypothesis is that ultrasonography muscle quantity measures (rectus femoris (RF) thickness and rectus femoris + vastus intermedius (RF+VI) thickness) and quality measures (Y–X index and echogenicity) are correlated with body composition measured by impedanciometry and handgrip strength and that a lower RF+VI and higher echogenicity are associated with a greater risk of hospital admission and death.

## 2. Materials and Methods

### 2.1. Design

This is an open, prospective observational study that analyzes the nutritional status of patients at the beginning of nutritional monitoring. Once the patients signed the informed consent and were included in this study, an anamnesis was conducted to collect data on affiliation, personal history, evolution of the disease, and nutritional history. In addition, anthropometry, bioimpedanciometry, handgrip strength, and muscle ultrasonography evaluations were performed. A record of medical nutritional therapy was taken both during the initial consultation and the patient’s follow-up. The results of all parameters were recorded in the same visit. 

This study was conducted between January 2021 and September 2022 in patients referred to the outpatient Clinical Nutrition Clinic at the University Clinical Hospital of Valladolid. The patient was followed up according to real-world clinical practice, and the data collection of evolution parameters was closed in January 2023.

### 2.2. Study Subjects

The inclusion criteria were patients with amyotrophic lateral sclerosis who were over 18 years old. The exclusion criteria were the patient’s inability to fully ambulate, neurodegenerative disease other than ALS, decompensated liver pathology, chronic kidney disease stage IV or higher, or failure to sign the informed consent by the patient. 

This study complied with the guidelines for human research established in the Declaration of Helsinki. This study was approved by the Medical Research Ethics Committee (CEIm) of the Health Area of Valladolid East with the code 22-2910; after that, the data collection process began. All patients had signed the informed consent prior to inclusion in this study. 

### 2.3. Variables

The variables collected were classified into clinical variables, morphofunctional assessment (anthropometric variables, body composition variables, and functional variables), and outcome variables. Clinical variables included age (years), sex (male/female), and type of amyotrophic lateral sclerosis (spinal/bulbar). 

The morphofunctional assessment included the following:

- Anthropometric variables: usual weight (kg), actual weight (kg), height (m), body mass index (actual weight/height x height (kg/m^2^)), arm circumference (cm), and calf circumference (cm).
- Electrical bioimpedanciometry (BIA) was performed with a bioimpedanciometer (BIA 101 Anniversary; EFG Akern). The BIA was performed between 8:00 and 9:15, after an overnight fast and after a time of 15 min in the supine position. The BIA measured the following raw electrical data: resistance (ohm), reactance (ohm), and phase angle (º). The following body composition parameters were estimated using validated formulas in Bodygram^®^ software, https://www.akern.com/en/products-and-solutions/data-analysis-software/bodygram-dashboard/ (Access data: 29 March 2024) (EFG, Akern, Pisa, Italy): fat mass index (FMI) (kg/m^2^), fat-free mass index (FFMI) (kg/m^2^), muscle mass index (MMI) (kg/m^2^), and body cell mass index (BCMI) (kg/m^2^). Appendicular skeletal muscle mass index (ASMI) (kg/m^2^) was measured by Sergi’s formula [10].
- Muscle ultrasonography (quadriceps rectus femoris (QRF) and vastus intermedius (VI)) of the dominant lower extremity was carried out with a 10–12 MHz probe and a multifrequency linear matrix (Mindray Z60, Madrid, Spain). The measurements of ultrasonography were made with the patient in a supine position [8]. The following muscle quantity parameters were measured: muscle area (cm^2^), muscle area index (muscle area/height × height) (cm^2^/m^2^), anteroposterior diameter of the QRF (*Y*-axis) (cm), transverse diameter of the QRF (*X*-axis) (cm), and the sum of the anteroposterior diameters of QRF and the vastus intermedius (RF+VI) (cm) [11].

The following muscle quality parameters were also determined: Y–X index (relation of the anteroposterior diameter (*Y*-axis) to the transverse axis (*X*-axis) of the QRF). On the other hand, the muscular echogenicity of the QRF was also determined, considering the transversal section of the muscle as a region of interest (ROI) as the area of QRF. The grayscale ranges from 0 (black) to 255 (white). The value is shown as a percentage (%) with respect to the maximum (255) [11]. The Image J^®^ program version 1.54 f (National Institutes of Health NIH, Bethesda, MD, USA) was used to determine echogenicity; this program is a method to treat radiological images developed by the National Health Institute (NIH) [12]. 

We considered muscle mass (RF+VI) and muscle quality (Y–X index) parameters as dichotomic variables. These variables were planted as low and high muscle mass: low RF+VI (male < 2.49 cm; female < 1.84 cm) and low Y–X index (<0.35). These values are obtained by the median of values in the sample stratified by sex. 

The diagnosis of malnutrition was made using the Global Leadership Initiative on Malnutrition (GLIM) criteria [13] through compliance with an etiological criterion and a phenotypic criterion. A 3-day nutritional survey was conducted to evaluate intake, and the patient’s nutritional requirements were evaluated using the Harris–Benedict equation [14]. The loss of muscle mass was measured with ASMI estimated by bioelectrical impedanciometry (low muscle mass: 7 kg/m^2^ in men and 5.5 kg/m^2^ in women) [15].

The outcome variables were the admission to a hospital ward or the death of the patient during the evaluation period (from inclusion in this study to the end of data collection). These variables were collected with data linkage to the regional electronic clinic history registry.

The variables studied as potential confounders were age, sex, clinical onset of amyotrophic lateral sclerosis, time from first symptoms to diagnosis, and time from diagnosis to evaluation in the Clinical Nutrition Unit. 

### 2.4. Data Analysis

Statistical analysis was performed with the SPSS 15.0 package (SPSS Inc., Chicago, IL, USA), officially licensed by the University of Valladolid. A normality analysis of continuous variables was performed using the Kolmogorov–Smirnov test. Normal continuous variables are expressed as mean (standard deviation) and non-normal continuous variables as median (interquartile range). The qualitative variables are represented with the number and percentage with respect to the total. 

Differences between parametric continuous variables were analyzed with the unpaired Student’s *t*-test, and differences between non-parametric variables were analyzed with the Mann–Whitney *U* test. A correlation analysis was performed to evaluate the relationship between the quantitative variables. A binary logistic regression was performed in a multivariate analysis to evaluate the relationship of the variables with the prognosis, and a survival analysis was conducted using log-rank and Kaplan–Meier curves. Qualitative variables will be expressed as percentages (%) and analyzed with the chi-squared test (with Fisher and Yates correction when it is necessary).

## 3. Results

### 3.1. Sample Description

A total of 37 patients diagnosed with amyotrophic lateral sclerosis (ALS) were included in this study. Of these patients, 51.4% were men. The mean age was 64.27 (12.59) years. A total of 54.1% of the patients had a bulbar onset of amyotrophic lateral sclerosis, and 45.9% had spinal onset. The median time between diagnosis of ALS and morphofunctional assessment was 10.5 (4–30) months. 

All patients with ALS followed in the Clinical Nutrition Unit in the study period were evaluated. All patients agreed to participate, and there was no abandonment. The loss of follow-up was caused by the death of patients. Five patients (13.5%) were unable to undergo the muscular ultrasonography due to their clinical state.

The morphofunctional assessment variables are shown in Table 1.

### 3.2. Malnutrition and Ultrasonography in ALS Patients

The percentage of subjects with malnutrition diagnosed by GLIM criteria was 45.9% of patients. There were differences in muscle mass parameters in ultrasonography but not in muscle quality parameters or fat tissue (Table 2). 

### 3.3. Ultrasonography and Other Body Composition Variables in ALS Patients

There was a direct correlation between BMI and Y–X index and between brachial circumference and Y–X index. There was a negative correlation between total muscle mass measured by rectus femoris and vastus internal (RF+VI) and the percentage of weight loss, as well as between brachial circumference and echogenicity (Table 3).

There was a direct correlation between active mass markers of bioimpedanciometry (BCMI, FFMI, and ASMI) and muscle mass parameters of nutritional ultrasonography (RF+VI and muscle area of rectus femoris index (MARFI)). If we measure the quality parameters of muscle ultrasonography (echogenicity and Y–X index), there was a direct correlation between echogenicity and resistance and between fat-free mass index and Y–X index, and there was a negative correlation between echogenicity and body cellular mass index (BCMI) and appendicular skeletal mass index (ASMI) (Table 4). There were no correlations in the female group due to the small sample size. 

**Figure 1:**
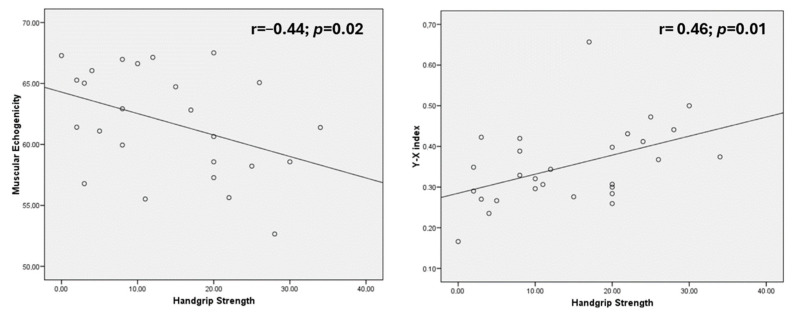
Relationships between muscle quality variables (echogenicity and Y–X index) and handgrip strength.

There was a direct correlation between handgrip strength and Y–X index (r = 0.46; *p* = 0.01); and RF+VI (r = 0.44; *p* = 0.02). There was a negative correlation between handgrip strength and echogenicity (r = −0.44; *p* = 0.02). There were no differences if it was stratified by sex (Figure 1).

### 3.4. Relationship between Morphofunctional Variables and Prognosis

In our sample, four patients (10.8%) died. There were 11 patients (29.7%) who needed admission to the hospital for any reason. The causes of admission were respiratory (four patients (10.8%)), digestive (four patients (10.8%)), and other causes (two patients (8.1%). 

There were no differences in muscle mass parameters of ultrasonography between patients who died and those who did not. The patients who died had more echogenicity (Table 5). In patients who were admitted for any reason, we observed low values of muscle mass parameters (*Y*-axis, RF+VI) and differences in muscle quality parameters (Y–X index and echogenicity) (Table 5).

We considered muscle mass (RF+VI) and muscle quality (Y–X index) parameters as dichotomic variables (low RF+VI (male < 2.49 cm; female < 1.84 cm); low Y–X index (<0.35)). Patients with low muscle (RF+VI) mass determined by ultrasonography showed an increased risk of admission adjusted by age, sex, and presence of dysphagia (OR: 7.84 (CI 95%: 1.09–56.07); *p*-value = 0.04), but no association was observed with the risk of death (OR: 2.89 (CI 95%: 0.22–37.84); *p*-value = 0.42). Patients with low-quality mass (Y–X index) had a higher risk of admission adjusted by age, sex, and presence of dysphagia (OR: 19.83 (CI 95%: 1.77–222.46); *p*-value = 0.02); however, they did not have an increased risk of death. 

**Figure 2:**
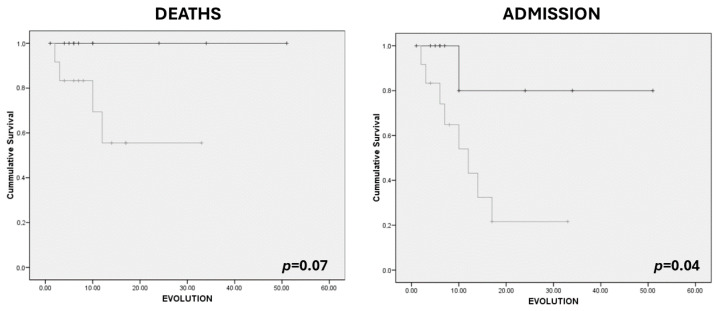
Kaplan–Meier curves for survival and risk of admission related to Y–X index in upper quartile patients (Q1, Q2) (black) (16 patients (43.2%)) vs. lower quartile (Q3, Q4) patients (grey) (16 patients (43.2%)).

**Figure 3:**
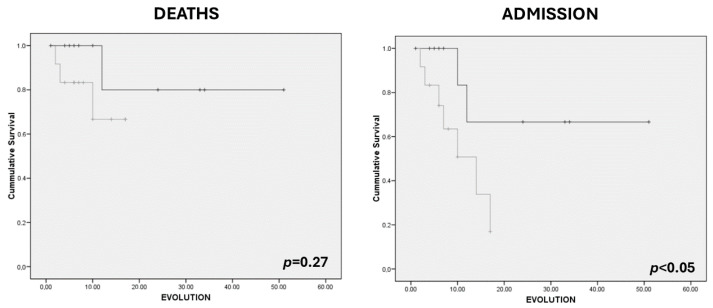
Kaplan–Meier curves for survival and risk of admission related to rectus femoris and vastus internal (RF+VI) in upper quartile patients (Q1, Q2) (black) (15 patients (40.5%)) vs. lower quartile (Q3, Q4) patients (grey) (17 patients (45.9%)).

Kaplan–Meier curves show an increase in admission in those who had a lower value of Y–X index (<0.35) (Figure 2) and an increase in admission in those who had a lower value of RF+VI (male < 2.49; female < 1.84) (Figure 3). There were no differences in survival.

## 4. Discussion

The patients with amyotrophic lateral sclerosis and malnutrition had lower values of anteroposterior diameter of muscle mass and lower parameters of muscle quality in muscle ultrasonography (MUS). There was an inverse correlation between weight loss and muscle mass by MUS, and there was a relation between body cellular mass index and muscle mass and quality parameters measured by MUS. Low muscle mass and quality determined by MUS had an increased risk for admission of any cause. 

Classical anthropometry in ALS is the most common parameter to diagnose malnutrition and to monitor the nutritional treatment. A study from Li et al. showed that patients with ALS had a lower value of body mass index (BMI) than controls (ALS: 23.52 (3.11) kg/m^2^; no ALS: 24.75 (3.34) kg/m^2^), and they had a median weight loss of 4.11% (−9.32–0.56) [16]. In our sample, BMI and weight loss were slightly higher than in this study. This condition could be based on the time from first symptoms to diagnosis and referral to the Clinical Nutrition Unit; in our case, this delay was 10.5 months. The early referral to the Clinical Nutrition Unit has shown a decrease in diagnosis of malnutrition in these patients [17]. A lower body mass index is related to poor survival in patients with ALS, and a BMI in high ranges can be protective for these patients, although weight gain or loss depends on the comorbidities of patients (nutritional status, respiratory function, or patients’ mobility) [18]. Body composition and its components could help us to select the better nutritional treatment for patients. 

In this study, body composition was assessed by bioelectrical impedanciometry and muscle ultrasonography. Bioelectrical impedanciometry could be used to measure body composition through the estimation of body compartments and body cellularity function and hydration through the electrical parameters. Patients in our sample had similar values of fat-free mass index (46.99 kg) to other studies with patients with ALS and higher values of fat mass index (22.09 kg); for example, compared to the Li et al. study (FFM: 47.83 kg; FMI: 17.42 kg) [16]. The phase angle was 5.02 (1.25)° in men and 5.26 (1.79)° in women; these values were lower than those from the general population of individuals of eighty or more years (men: 5.3° (IC95%: 4.5–6.0); women: 5.4° (IC95%: 5.3–5.6)) [19]. These parameters show the changes in body composition that could be masked by a normal BMI, and they can be related to the patient’s prognosis [20]. Functional assessment by handgrip strength showed a significant decrease in patients with respect to the general population [21] and the sample with ALS from Musaro et al., which showed a mean handgrip strength of 22 (6.2) kg [22]. 

Quadriceps ultrasonography is a novel technique to assess body composition (muscle and fat mass) in patients at risk of malnutrition to diagnose malnutrition and sarcopenia and evaluate prognosis. This technique has not been well studied in patients with ALS, but it has been studied in other individuals at risk of malnutrition as older patients after hip fracture (quadriceps thickness (RF+VI): 2.22 (0.65) cm) [23] or patients with stroke with normal nutritional status (quadriceps thickness: paralytic, 3.32 cm; non-paralytic, 3.45 cm) [24]. The values are higher than those of our sample in relation to the muscle progression of the disease. Values of muscle quality cannot be compared because they are not measured, or the measurement of echogenicity was not performed with the same method we used. 

The ESPEN guidelines in neurology recommend screening for malnutrition (BMI and weight loss) at diagnosis and during the follow-up [5]. In patients with amyotrophic lateral sclerosis, body composition can be related to these low values of BMI, especially in patients with <18.5 kg/m^2^; these data have been seen in the Barone et al. study in patients who had undergone percutaneous endoscopic gastrostomy (PEG), which showed low values of body cellular mass and phase angle in underweight patients [25]. This relation cannot be clearly diagnosed in patients with higher values of BMI without weight loss. In a previous study from our group, 48.4% of patients had malnutrition according to the GLIM criteria, and 71% were at risk or with malnutrition according to subjective global assessment (SGA); these differences could be related to the difficult assessment of muscle mass [3]. Muscle ultrasonography can be a useful tool to assess malnutrition in patients with no weight loss or low BMI. This study showed lower values of muscle quantity (rectus femoris thickness (*Y*-axis) and quadriceps thickness (RF+VI)) and lower values of muscle quality (Y–X index) without changes in muscle area. The values observed in patients with malnutrition were similar to those parameters of another study of ultrasonography in patients with malnutrition [11].

Muscle ultrasonography is an adequate tool to diagnose muscle mass and quality in patients with disease-related malnutrition. Nevertheless, amyotrophic lateral sclerosis has some clinical issues that affect the development of malnutrition. Muscle mass parameters measured by ultrasonography (quadriceps thickness) have an inverse relationship with the percentage of weight loss, as has been demonstrated in the study by Li et al. [16]. On the other hand, ultrasound muscle mass (quadriceps thickness and muscle area) parameters have an inverse relationship with resistance and a direct relationship with estimated muscle mass parameters defined by impedanciometry; this condition demonstrates a relationship of leg muscle mass with variables that estimate whole body muscle mass. This relationship could be useful for measuring active mass and monitoring nutritional treatment in patients with ALS, as Tandan et al. demonstrated with DXA values [26]. Muscle quality determined by ultrasonography (Y–X index and echogenicity) had a direct relationship with handgrip strength; this circumstance has been observed in other patients with disease-related malnutrition with an inverse relationship with echogenicity (r = −0.36) and X–Y index (r = −0.18). This issue is more interesting than muscle mass because disease progression could be related to a false diagnosis of malnutrition, but the conservative effect on muscle function can be measured by muscle quality ultrasonography, and this could be a good variable to see changes in rehabilitation and nutritional treatment. Until now, the impact of rehabilitation and nutritional treatment on muscle functionality could only be assessed through electrophysiology or handgrip strength, which have limitations in diagnosis [22].

Energy deposits are very important in patients with ALS; in fact, low fat mass measured by computed tomography (CT) is an independent poor prognostic factor for survival [27]. Another study by Lee et al. showed that a low baseline percentage of body fat correlated with a decline in quality-of-life tests (r = 0.72) [28]. In our sample, subcutaneous adipose tissue correlated with resistance and reactance, but a negative relationship with estimated fat-free mass index or appendicular skeletal mass index; this condition could be related to higher values of resistance and reactance in patients with higher BMI and diagnosis of obesity. However, in our sample, there were no differences in fat mass between patients with comorbidities and those without.

Multivariate analysis showed an increase in hospital admission risk in patients in lower quartiles of muscle mass. This situation can be related to differences in complications associated with ALS in patients with a lower muscle mass. Salvini et al. demonstrated that changes in body composition in ALS were related to the risk of dysphagia [29], and De Vito et al. saw that compartmental nutritional evaluation could be related to respiratory muscle function [30]. There were no differences in survival analysis for death; this condition must be related to the observation time of patients (2 years) and the variability in the course of the disease. Muscle quality parameters have shown similar results, with an increase in admission in lower quartiles and no relation to survival. These data resemble those of patients with disease-related malnutrition and a high X–Y index (instead of the low Y–X index of this study), which demonstrate an increase in the number of deceased patients in the highest quartiles of the X–Y index [11].

The main strength of this study is the use of morphofunctional assessment in a sample of patients with ALS; in these cases, the use of anthropometry and body composition is very difficult, and the development of new methods such as MUS is very important for diagnosing malnutrition and monitoring medical nutrition treatment. Another strength of this study is the use of parameters different than anthropometric to evaluate prognosis in these patients. The main limitation of this study is the small sample size in relation to the low incidence and prevalence of this disease; this fact can influence the dispersion of any results and minimize the changes. Another limitation is the differences in progression between patients related to the physiopathology of the disease; this condition can influence the results on survival rate, but it does not interfere with the correlation or malnutrition analysis. Although there was a significant difference in some variables between males and females, it was not possible to stratify the correlation study between sexes due to the small sample size; however, in the multivariate analysis of prognostic factors, we have included sex to adjust the hazard ratio as a potential confounder.

The future lines of investigation are related to the relationship between nutritional ultrasonography and the diagnosis of malnutrition in patients with ALS in multicenter studies. For this reason, the development of cut-off points for malnutrition and sarcopenia in these patients and persons with no disease is needed. It would also be interesting to know the role of ultrasonography in monitoring nutritional status and medical nutrition therapy in patients with ALS. Further studies with a larger sample size and intervention design will be necessary to increase knowledge on this topic.

## 5. Conclusions

Baseline morphofunctional assessment of patients with amyotrophic lateral sclerosis shows low values of phase angle and handgrip strength parameters with respect to the normal population. Muscle ultrasonography parameters have lower values with respect to other samples of patients with disease-related malnutrition. In patients with ALS and malnutrition, muscle mass parameters (quadriceps thickness and rectus femoris thickness) and muscle quality parameters (Y–X index) are lower than those of patients without malnutrition.

Muscle mass parameters in ultrasonography in patients with ALS are related to electric parameters, and estimated muscle mass parameters (FFMI, ASMI, and BCMI) are related to impedanciometry. Ultrasonography echogenicity is inversely related to BCMI, FFMI, and ASMI, and the Y–X index is directly related to FFMI.

Muscle ultrasonography could be a prognostic factor in patients with ALS. The lowest quartiles of quadriceps thickness and Y–X index are risk factors for admission, but there was no association for death risk.

## Figures and Tables

**Table 1:** Morphofunctional assessment variables and differences between sexes.

	Total	Men	Women	*p*-Value
Anthropometry				
BMI (kg/m^2^)	26.52 (4.51)	27.46 (4.54)	25.45 (4.37)	0.20
%weight loss	5.29 (6.07)	4.74 (4.70)	6.06 (7.76)	0.59
Arm circumference (cm)	27.09 (2.27)	27.58 (2.11)	26.56 (2.37)	0.20
Calf circumference (cm)	34.08 (3.91)	34.53 (4.08)	33.59 (3.78)	0.49
Bioelectrical Impedanciometry				
Resistance (ohm)	575.24 (112.07)	508.33 (64.33)	655.53 (104.98)	<0.01
Reactance (ohm)	50.64 (15.41)	44.06 (10.57)	58.53 (16.88)	<0.01
Phase angle (°)	5.13 (1.50)	5.02 (1.25)	5.26 (1.79)	0.66
ASMM (kg)	17.57 (4.00)	20.48 (2.42)	14.07 (2.36)	<0.01
ASMI (kg/m^2^)	6.53 (1.03)	7.12 (0.74)	5.82 (0.89)	<0.01
FFM (kg)	46.99 (10.07)	54.48 (6.75)	38.01 (4.23)	<0.01
FFMI (kg/m^2^)	17.45 (2.15)	18.89 (1.57)	15.72 (1.33)	<0.01
BCM (kg)	22.82 (6.99)	26.31 (6.41)	18.62 (5.24)	<0.01
BCMI (kg/m^2^)	7.97 (2.08)	8.56 (1.91)	7.27 (2.12)	0.08
FM (kg)	22.09 (8.26)	21.63 (7.61)	22.65 (9.21)	0.73
FMI (kg/m^2^)	8.38 (3.25)	7.61 (3.00)	9.31 (3.40)	0.14
Muscle Ultrasonography				
MARFI (cm^2^/m^2^)	1.29 (0.51)	1.44 (0.57)	1.14 (0.41)	0.09
*Y*-axis (cm)	1.17 (0.35)	1.29 (0.38)	1.03 (0.25)	0.03
*X*-axis (cm)	3.27 (0.63)	3.47 (0.59)	3.05 (0.61)	0.06
RF+VI (cm)	2.18 (0.68)	2.49 (0.66)	1.84 (0.54)	<0.01
SCAT (cm)	1.07 (0.72)	0.59 (0.31)	1.37 (0.41)	<0.01
Y–X index	0.36 (0.09)	0.37 (0.10)	0.34 (0.09)	0.41
Echogenicity (%)	61.77 (4.63)	61.67 (4.49)	61.86 (4.29)	0.91
MUSCLE STRENGTH				
Handgrip strength (kg)	14.54 (9.67)	16.81	11.50	0.15

BMI, body mass index; ASMM, appendicular skeletal muscle mass; ASMI, appendicular skeletal mass index; FFM, fat-free mass; FFMI, fat-free mass index; BCM, body cell mass; BCMI, body cell mass index; FM, fat mass; FMI, fat mass index; MARFI, muscle area rectus femoris index (cm/m^2^). X, transversal rectus femoris axis; Y, anteroposterior rectus femoris axis; RF+VI, rectus femoris + vastus internal; SCAT, subcutaneous adipose tissue.

**Table 2:** Differences in ultrasonography parameters by diagnosis of malnutrition.

	Malnutrition	No Malnutrition	*p*-Value
Sex (male/female)	52.9%/47.1%	56.3%43.8%	0.72
MARFI (cm^2^/m^2^)	1.13 (0.49)	1.46 (0.52)	0.08
*Y*-axis (cm)	0.99 (0.32)	1.37 (0.27)	<0.01
*X*-axis (cm)	3.24 (0.68)	3.33 (0.60)	0.69
RF+VI (cm)	1.81 (0.64)	2.61 (0.46)	<0.01
SCAT (cm)	0.95 (0.45)	0.94 (0.63)	0.96
Y–X index	0.30 (0.06)	0.42 (0.09)	<0.01
Echogenicity (%)	62.94 (4.87)	60.43 (4.29)	0.17

MARFI, muscle area rectus femoris index (cm/m^2^); X, transversal rectus femoris axis; Y, anteroposterior rectus femoris axis; RF+VI, rectus femoris + vastus internal; SCAT, subcutaneous adipose tissue.

**Table 3:** Correlation between ultrasonography and parameters of anthropometry assessment.

	RF+VI	MARFI	Echogenicity	Y–X Index
%WL	r = −0.42; *p* = 0.04 *	r = −0.15; *p* = 0.47	r = 0.04; *p* = 0.85	r = −0.37; *p* = 0.07
BMI (kg/m^2^)	r = 0.33; *p* = 0.06	r = 0.05; *p* = 0.78	r = −0.04; *p* = 0.83	r = 0.40; *p* = 0.02 *
Braquial circumference (cm)	r = 0.34; *p* = 0.06	r = 0.17; *p* = 0.36	r = −0.43; *p* = 0.02 *	r = 0.37; *p* = 0.04 *
Calf circumference (cm)	r = 0.34; *p* = 0.06	r = 0.26; *p* = 0.14	r = −0.27; *p* = 0.16	r = 0.18; *p* = 0.33

%WL, percentage weight loss; MARFI, muscle area of rectus femoris index; RF+VI, rectus femoris + vastus internal; BMI, body mass index; * *p* < 0.05.

**Table 4:** Correlation between ultrasonography and parameters of bioelectrical impedanciometry.

	RF+VI	SCAT	MARFI	Echogenicity	Y–X Index
Resistance (ohm)	r = −0.59; *p* < 0.01 *	r = 0.59; *p* < 0.01 *	r = −0.5; *p* < 0.01 *	r = 0.44; *p* = 0.02 *	r = −0.19; *p* = 0.28
Reactance (ohm)	r = −0.06; *p* = 0.72	r = 0.42; *p* = 0.02 *	r = 0.24; *p* = 0.19	r = −0.07; *p* = 0.72	r = −0.05; *p* = 0.76
Phase angle (°)	r = 0.28; *p* = 0.11	r = 0.04; *p* = 0.83	r = 0.54; *p* < 0.01 *	r = −0.30; *p* = 0.10	r = 0.07; *p* = 0.71
BCMI (kg/m^2^)	r = 0.62; *p* < 0.01 *	r = −0.26; *p* = 0.16	r = 0.64; *p* < 0.01 *	r = −0.46; *p* < 0.01 *	r = 0.27; *p* = 0.14
FFMI (kg/m^2^)	r = 0.75; *p* < 0.01 *	r = −0.53; *p* < 0.01 *	r = 0.52; *p* < 0.01 *	r = −0.38; *p* = 0.04 *	r = 0.36; *p* < 0.01 *
FMI (kg/m^2^)	r = −0.04; *p* = 0.83	r = 0.59; *p* < 0.01 *	r = −0.17; *p* = 0.34	r = 0.25; *p* = 0.19	r = 0.26; *p* = 0.15
ASMI (kg/m^2^)	r = 0.69; *p* < 0.01 *	r = −0.42; *p* = 0.02 *	r = 0.58; *p* < 0.01 *	r = −0.40; *p* = 0.03 *	R = 0.34; *p* = 0.06

MARFI, muscle area of rectus femoris index; RF+VI, rectus femoris + vastus internal; SCAT, subcutaneous adipose tissue; * *p* < 0.05.

**Table 5:** Differences in nutritional ultrasonography in relation to the admission and death.

	Death	No Death	*p*-Value	Admission	No Admission	*p*-Value
Gender (M/F)	5.3/16.7	94.7/83.3	0.34	21.1/38.9	78.9/61.1	0.29
Nutritional Ultrasonography						
MARFI (cm^2^/m^2^)	1.28 (0.27)	1.29 (0.54)	0.97	1.07 (0.46)	1.40 (0.52)	0.08
*Y*-axis (cm)	1.03 (0.22)	1.19 (0.36)	0.39	0.95 (0.28)	1.27 (0.33)	0.01
*X*-axis (cm)	3.44 (0.36)	3.25 (0.66)	0.57	3.31 (0.61)	3.26 (0.65)	0.83
RF+VI (cm)	1.66 (0.30)	2.26 (0.69)	0.09	1.65 (0.44)	2.42 (0.64)	<0.01
SCAT (cm)	0.97 (0.54)	0.96 (0.54)	0.97	1.01 (0.43)	0.94 (0.57)	0.71
Y–X index	0.29 (0.06)	0.37 (0.10)	0.19	0.28 (0.06)	0.39 (0.09)	<0.01
Echogenicity (%)	66.92 (3.03)	61.28 (5.26)	<0.05	64.90 (4.05)	60.59 (5.43)	0.03

M, male/F, female; MARFI, muscle area of rectus femoris index; RF+VI, rectus femoris + vastus internal; SCAT, subcutaneous adipose tissue.

## Data Availability

Data are unavailable due to privacy or ethical restrictions.
